# Bifactor modelling and measurement invariance testing of the Innovative Behaviour Inventory

**DOI:** 10.4102/ajopa.v5i0.113

**Published:** 2023-08-31

**Authors:** Hamfrey Sanhokwe, Willie Chinyamurindi, Joe Muzurura

**Affiliations:** 1 Graduate School of Business Leadership, Faculty of Business Sciences, Midlands State University, Gweru, Zimbabwe; 2 Department of Business Management, University of Fort Hare, East London, South Africa; 3 Department of Economics, Faculty of Business Sciences, Midlands State University, Harare, Zimbabwe

**Keywords:** desirable behaviour, reliability, validity, dimensionality, measurement invariance, periodic corporate assessments, targeted interventions

## Abstract

Innovation-driven growth demands that organisations periodically assess the innovative behaviour of employees and facilitate appropriate interventions to nurture it. Human behaviour is complex. Corporate researchers rely on standard measures to appreciate employee behaviours at work. Quality tools support decision-making by detecting gaps and creating opportunities for improvement at work. One promising measure is the Innovative Behaviour Inventory (IBI). Although the IBI was developed and validated in Europe, it can still be applied in other contexts if it exhibits adequate psychometric properties and diagnostic utility. Using a probability sample drawn from Zimbabwe, the study used bifactor analysis to assess the dimensionality of the IBI measurement model. The study assessed the measurement invariance of the IBI using multi-group confirmatory factor analysis. The study modelled the general factor of the IBI. Multiple tests confirmed the internal consistency reliability, convergent and discriminant validity of the measure. The IBI was invariant across gender groups. The IBI adds to the suite of available tools for assessing innovative work behaviour as part of a dedicated leadership excellence agenda. The IBI offers practical advantages because of its integrated, multi-faceted nature, diagnostic utility and robust psychometric properties. Periodic surveys, using the IBI, provide corporate researchers and organisational leaders with information on the quality of innovative behaviour that resides in their organisations. The IBIs invariance and ability to quantify, as summative scores, the levels of innovative behaviour enable sub-group analysis. Sub-group analytical outputs facilitate targeted interventions in the workplace.

**Contribution:**

Appreciating the quality of the IBI measurement model carries ethical and practical significance. Studies of this nature safeguard the subjects of research and promote purpose-driven corporate and scholarly work.

## Introduction

The continued disruption of the workplace calls for continuous innovation if organisations are to remain sustainably future-fit (Volberda et al., 2018). The role of employees in the growth and development of organisations has been a subject of corporate and academic research interest over the last century, albeit slowly. The need to sustainably generate new sources of value has reinvigorated interest in appreciating the employee-driven innovation process. Given the growing interest in developing and sustaining workplace innovations, accurate, ethically sound, and sustainable assessment of employee innovative behaviour has become a critical organisational excellence issue.

### Historical development of the ‘innovation’ concept

Since time immemorial, societies have relied on implementing ideas as improvements or solutions to systems, extant processes and products to address current or emerging problems, if not needs and wants (Godin & Vinck, 2017).

Various schools of thought exist regarding how the term innovation came into being. According to Godin (2015), the term innovation is derived from two seemingly contrasting terms, invention and imitation, derived from Greek philosophy. Literature traces the imitation of reality to the work of Plato (Taylor, 2017). The imitation of goods and services to improve their quality, design and appearance is a theme that dates back to the 16th century and continues to this day.

From an invention perspective, the literature emphasises important episodes dating back to the 14th century that places scientific discovery and the search for the ‘new’ at the heart of early efforts that culminated in the industrial revolution (Taylor, 2017). The shift to the economics of profit, which continues to impact society, is seen as a massive driver of the inventions in the 19th century. During this time, Marx introduced the economic theory of social and technological advances and the conceptualisation of production efficiencies (Godin, 2015).

In the early to mid-20th century, innovations were conceived as instruments of survival for firms and economic growth; hence, the attention on the same grew (Taylor, 2017). By the late 20th century, the innovation terminology had morphed and embedded technological change and societal advancement and development, albeit the different interpretations of the same (Taylor, 2017). Since the dawn of the new millennium, the term ‘innovation’ has assumed myriad meanings and conceptualisations, with a growing desire to appreciate the factors influencing the phenomenon.

### Defining the concept of innovation

A closer introspection of the available literature shows a wide array of definitions of the phenomenon, primarily influenced by the field of the study, sector and whether one is looking at products, services and/or processes. These conceptualisations enrich understanding of the phenomenon despite the various languages and interpretations of the same (Taylor, 2017). The available literature details the constituent elements of the concept, as well as the models, frameworks and theories that underpin the concept.

Schumpeter (1934, 1942) acknowledged and documented the vital role of innovation in the 1930s and early 1940s. The conceptualisation by Schumpeter emphasised the creation or production of new combinations from the existing, yet distinct, resources or elements. The Organisation for Economic Co-operation and Development (OECD, 2005) International Guidelines embed the ‘new’ and ‘improvements’ in defining innovation. The OECD guidelines go beyond products and processes to include new and/or improved changes in marketing, workplace organisation (including management and skills) and relations (also see De Vires et al., 2014). The Confederation of British Industry (CBI/QinetiQ) broadened the definition, that is, innovation pertained to the transformation of behaviours and ideas into sources of value (Taylor, 2017; also see Damanpour & Schneider, 2009). Such transformations include the practical application of ideas to enhance business models that alter, in a progressive fashion, the ways and approaches to working (see Taylor, 2017).

While the above conceptualisations appeared more inward looking, Evers et al. (2014) were more explicit in highlighting the disruptive nature of innovations to present structures and routines internal and external to the organisation. Dogru and Peyrefitte (2022), building on extant literature, defined innovations as novel solutions of practical, financial and/or social significance or value. In unison, the above conceptualisations emphasise two critical features of innovations, that is, offering solutions to existing or emerging practical needs and adding value to stakeholders and shareholders alike.

Viewed this way, innovations are value creating and adding actions. Such actions can be viewed as an outcome (hence the need to understand how to influence them), a process (the focus is on how the actions are organised optimally) and as a mindset (role of individuals and the organisational environment as important influence levers) (Kahn, 2018). An essential attribute of contemporary research is the inclination towards appreciating the role of individuals in the innovation process (Abd Awang et al., 2019).

### Historical development of the innovative work behaviour construct

During the 20th century, literature shifted towards the need to understand *who* was responsible for innovation. Researchers took an active interest in understanding the psychological aspects inherent in the innovation process. Researchers began to develop linear models to explain what motivated the acts of innovation. Simonton (1984) reckoned the role of individual efforts and dedication as a driver of innovation. West and Farr (1990) further articulated how innovations appeared to exist at multi-levels, that is, individual, and accumulating at the group and organisational levels.

The submission by West and Farr (1990) was further supported by Scott and Bruce (1994) who suggested that for innovations to be enjoyed at the organisational level, they should be inculcated at the individual level. The study by Scott and Bruce (1994) also raised an important finding: there was an apparent lack of a correlation between the level of education and dimensions of innovation. From a workplace perspective, this introduced the need for an enterprise-wide focus on innovative behaviour regardless of the level of work.

### Defining employee innovative behaviour

Employees interact with organisational processes, services and products on an ongoing basis. Through such interactions, employees can presumably recognise problems, detect performance gaps, explore opportunities and seek new or alternative ways of doing things (Bos-Nehles et al., 2017). Extra-role behaviours and capabilities anchor the proactive generation, introduction and application of innovative ideas on specific aspects of work in a manner that creates value, attains advantage and ensures work sustenance for the benefit of shareholders and stakeholders alike (AlEssa & Durugbo, 2022). It is a choice to act proactively for the organisation’s good and/or to satisfy self-serving interests.

Employee innovative behaviour is a complex, value-creating behaviour that resides in and is self-initiated by employees. Such behaviour helps identify potential opportunities and solutions and develops new procedures and methods that benefit the organisation (AlEssa & Durugbo, 2022).

Innovative work behaviour creates new or improved dimensions to business conduct and performance. The behaviour gives organisations a new impetus by renewing and revitalising various aspects of the work and workplace – including processes, products and procedures – in a goal-directed manner (AlEssa & Durugbo, 2022). The created value can be incremental or radical.

Most scholars present innovative behaviour as a multi-dimensional phenomenon (De Jong & Den Hartog, 2010; Lukes & Stephan, 2017; Scott & Bruce, 1994). For instance, Scott and Bruce (1994) operationalised it as encompassing idea generation, coalition building and implementation. Janssen (2000) depicts three elements: generation of ideas, promotion of ideas to colleagues and management and realising the ideas. Kleysen and Street (2001), on the other hand, present five elements: exploration of the opportunity, generativity, investigation or interrogating the available and missing information, championing the ideas and application of the ideas. The submissions by De Jong and Den Hartog (2010) point to four aspects, namely the exploration of ideas, generation of succinct thoughts and ideas (entails the processing of the ideas), championing the ideas that seem to add value and implementation of the select ideas. The recent submission by Lukes and Stephan (2017), the basis for this study, recognises six elements: generation of ideas, searching for noble, contextual ideas, communicating the ideas to constituencies that matter (colleagues, leadership, others), implementation starting activities, involving significant others and overcoming any real or perceived obstacles.

Two main theoretical perspectives dominate contemporary research on the voluntary activation of innovative behaviour. These are the individualistic and intrapreneurial perspectives.

#### The individualistic perspective

This perspective is delineated into two, that is, the behaviourists and the Gestalt-field psychologists (Ibrahim et al., 2015; Taylor, 2017). The behaviourist view depicts behaviour as an outcome of the interaction between the employee and their environment. Behaviourists assume that the activation of a specific behaviour is influenced by the expected consequences (Taylor, 2017). In this vein, reward mechanisms are fundamental in shaping employee behaviour. Gestalt-field psychologists submit that employee behaviour is influenced by how individuals use reason in interpreting the stimuli from the environment to which they are exposed (Ibrahim, 2015). In other words, the observed behaviour is an outcome of the environment and reason.

One of the core theories under behaviourism is the expectancy theory, anchored on cognitivism. The theory emphasises the role of external motivation and how perceptions of expectations (e.g. positive incentives or lack thereof) activate behaviours towards the desired outcome (Taylor, 2017). For example, an employee chooses (implying there are alternatives) a specific behaviour simply because of what they expect their behaviour to yield; that is, the desirability of an outcome fosters the activation of a certain behaviour(s).

Ryan and Deci (2019, 2020) do not submit to the external motivation school of thought. Instead, they argue that self-motivation is central to behaviour and responsibility; see self-determination theory. Intrinsic motivation implies that an employee is wholly involved in the activity, and the reward is the feeling of accomplishment and enjoyment (Ryan & Deci 2019, 2020). The submissions by Ryan and Deci (2019, 2020) resonated with earlier scholars such as Bandura (1977). According to Bandura (1977), individuals possess self-directing capacities and capabilities. This submission from a seminal study by Bandura (1977) views employees as active agents who interpret and reciprocally interact with their environment. Bandura (1977) submitted that images of desired futures by employees encourage thoughts and actions towards the desired distant goals.

#### The intrapreneurial perspective

The intrapreneurial perspective offers a complementary vantage point in explaining the activation of employee innovative behaviour. As highlighted earlier, employees enjoy exclusive insights into a company’s daily processes (Bos-Nehles et al., 2017). Additionally, virtual and in-person exposure to the workplace enhances their understanding of their needs and customer expectations (Bos-Nehles et al., 2017). The perspectives of individuals and the group are critical for producing ideas and executing effective problem solutions. Intrapreneurship is an approach pinned on collaborative, inclusive, internally networked cultures that contribute to organisational goals.

The intrapreneurship perspective places value on the characteristics of the employee while acknowledging the multilevel, cross-organisational interaction that happens in an iterative way to produce value (Blanka, 2019; Duradoni & Di Fabio, 2019). This perspective is anchored on the recognition that businesses operate in very fluid environments where long-range technical and strategic planning is complex. Therefore, the primary function of leadership is to develop and nurture environments that promote individual employee, group-level and organisational learning as well as other conditions that encourage adaptation and strategic agility.

The theoretical perspectives articulated above provide useful insights on how to model this desirable behaviour in the workplace. The workplace remains complex; hence, the integrated perspectives articulated above broaden possibilities and opportunities for influencing the behaviour.

### Limitations of the innovative work behaviour construct

The construct presupposes employees to be rational actors in their pursuit of personal and/or organisational goals. Research on employee innovative behaviour does not presume that these acts benefit the organisation *per se*. The construct largely emphasises the employee’s perspective. Employee innovative behaviour could be detrimental or counterproductive to the organisation or viewed in a bad light by those in leadership. Hence, open lines of communication are needed to ensure alignment and course corrective actions, as necessary.

Research on innovative work behaviour largely carries an intrinsic motivational bias, that is, employees desire to experience challenging work to develop their skills and competencies (Phillips, 2021). However, from an organisational perspective, how the employee acts of innovation accumulate into value-adding resources over time cannot be predicted with certainty. This argument could result from the difficulty of tracking and monitoring incremental innovation activities at work. Unlike radical innovations that are easily measurable, for example, through patents, the same cannot be said of incremental innovations. While contemporary research emphasises the use of teams, including integration of the same, the innovative work behaviour construct only explains how and why employees act voluntarily in an innovative fashion in their capacities and based on their perceptions.

Despite these limitations, the need for organisations to remain sustainably future-fit in the presence of change challenges leaders and followers alike to reimagine how best to influence and support this behaviour continuously.

#### Measuring employee innovative behaviour

Employee innovative behaviour (also known as innovative work behaviour) remains an invaluable concept in organisational and work psychology. Firstly, innovative work behaviour is indicative of job satisfaction (Al-edenat, 2018), psychological safety (Edmondson, 2018), leadership qualities (Amankwaa et al., 2022), perceived organisational support and workplace practices (Kwon & Kim, 2020; Le & Lei, 2019) as it is promoted by such conditions. Secondly, innovative work behaviour predicts organisational growth and development outcomes (Musneh et al., 2021). Thirdly, innovative work behaviour is an important variable when challenges such as organisational inertia and mal-competitiveness (Kiveu et al., 2019), discontinuation of business (Sanhokwe, 2022a), or low employee, organisational and societal wellbeing (Griffith, 2021; Kahn, 2018) emerge in the workplace because the quality of innovations is antecedent to such problems.

According to the Global Innovation Index (GII) report (2022), only a small subset of economies and organisations have delivered peak innovations consistently over time (WIPO, 2022). Sub-optimal innovations constrain economic and general development. At the organisational level, innovation stagnation curtails organisational performance and value generation (Sanhokwe, 2022a). Effectively assessing the innovation capabilities of employees, on an ongoing basis, across all levels, provides organisations with timely information on the quality of this dynamic capability. Without adequately understanding the employee innovative behaviour construct, including the mechanisms and pathways to influence it, organisational efforts to sustainably generate new sources of value are constrained. When done in an ethically correct manner, using standard measures, these assessments assist organisations in developing systematic, sustainable and targeted interventions that promote the desired behaviour.

One promising instrument for assessing innovative work behaviour is the Innovative Behaviour Inventory (IBI) developed by Lukes and Stephan (2017). Although the IBI was developed and validated using a multi-country sample from the Czech Republic, Germany, Italy and Switzerland, it can still be applied in other contexts if it exhibits adequate psychometric properties and diagnostic utility. Lukes and Stephan (2017) reckoned the need to explore whether the IBI could be employed in non-European organisational settings.

The IBI is publicly available and could serve as an ideal measure for assessing innovative behaviour in global south work contexts. Doing so requires an evaluation of the IBI measurement model in the contextual setting of interest, the crux of this study. The study tested the psychometric properties of the IBI and evaluated its utility using a sample drawn from the manufacturing sector in Zimbabwe.

### The Innovative Behaviour Inventory

The IBI is an integrated six-factor, 20-item, reflective measure designed to map the multi-faceted nature of the innovative behaviour construct (Lukes & Stephan, 2017). Built on earlier conceptualisations by scholars such as De Jong and Den Hartog (2010), Janssen (2000), Kleysen and Street (2001), Scott and Bruce (1994), Zhou and George (2001), the IBI is a recently developed, self-report measure. Employees self-rate their perceived levels of innovative behaviour using a five-point Likert-type scale (from fully disagree [1] to fully agree [5]). A higher score denotes a perceivably greater inclination in that behavioural attribute. For this study, we used the seven-point Likert-type scale. Compared to others, a seven-point Likert scale has been shown to exhibit higher accuracy and offers a better evaluation of the behavioural aspects of interest (see Taherdoost, 2019).

The IBI comprises six dimensions namely idea generation, idea search, idea communication, implementation starting activities, involving others and overcoming obstacles. Idea generation denotes the behavioural component of creativity (Lukes & Stephan, 2017). Idea search depicts the proactive exploration for new or existing knowledge by tapping into internal or external sources (Lukes & Stephan, 2017). Idea communication emphasises the role of engaging and seeking feedback; typically, this culminates in the formal or informal (dis)approval of ideas or concepts (Lukes & Stephan, 2017). Idea implementation is the transformation of abstract concepts into practice (Lukes & Stephan, 2017). Idea implementation is a resource consuming act (time, employees as general and dynamic capabilities and money), led by a defined point of contact. Organisations consist of talent with myriad skills and capabilities. The quality of execution depends on the extent to which myriad general and dynamic capabilities are harnessed for the good of the organisation (involving others; Lukes & Stephan, 2017). Today’s business environment is laden with challenges; exploring ways to overcome them is critical and contingency measures are required (Lukes & Stephan, 2017).

Since its development, the IBI has not been altered. The 20 items of the IBI are presented in Table 1. The IBI has exhibited robust psychometric properties in previous studies in Europe. Specifically, studies have reported acceptable reliability (Cronbach *α* values ranging from 0.60 to 0.93 for the six subscales, see Lukes & Stephan, 2017; 0.92 for the full scale, see Wiener & Sedger, 2021). Lukes and Stephan (2017) also reported the criterion, factorial, convergent and discriminant validity of the IBI. Besides the original study by Lukes and Stephan (2017), there is scant information on the quality of the IBI measurement model in non-European settings. This is despite the potential the measure holds in assessing the construct and facilitating remediation efforts. The original factor analytic test by Lukes and Stephan (2017) presents the IBI as a second-order factor comprising six first-order factors.

**TABLE 1:** Innovative Behaviour Inventory sub-scales, items and related factor loading.

Item	Factor loading
**Idea generation**	
‘I try new ways of doing things at work’.	0.449
‘I prefer work that requires original thinking’.	0.847
‘When something does not function well at work, I try to find new solution’.	0.621
**Idea search**	
‘I try to get new ideas from colleagues or business partners’.	0.808
‘I am interested in how things are done elsewhere in order to use acquired ideas in my own work’.	0.750
‘I search for new ideas of other people in order to try to implement the best ones’.	0.967
**Idea communication**	
‘When I have a new idea, I try to persuade my colleagues of it’.	0.744
‘When I have a new idea, I try to get support for it from management’.	0.902
‘I try to show my colleagues positive sides of new ideas’.	0.930
‘When I have a new idea, I try to involve people who are able to collaborate on it’.	0.773
**Implementing starting activities**	
‘I develop suitable plans and schedules for the implementation of new ideas’.	0.782
‘I look for and secure funds needed for the implementation of new ideas’.	0.847
‘For the implementation of new ideas I search for new technologies, processes or procedures’.	0.842
**Involving others**	
‘When problems occur during implementation, I get them into the hands of those who can solve them’.	0.869
‘I try to involve key decision makers in the implementation of an idea’.	0.928
‘When I have a new idea, I look for people who are able to push it through’.	0.710
**Overcoming obstacles**	
‘I am able to persistently overcome obstacles when implementing an idea’.	0.658
‘I do not give up even when others say it cannot be done’.	0.943
‘I usually do not finish until I accomplish the goal’.	0.884
‘During idea implementation, I am able to persist even when work is not going well at the moment’.	0.646

*Source*: Adapted from Lukes, M., & Stephan, U. (2017). Measuring innovative: A review of existing scales and the development of the innovative behaviour and innovation support inventories across cultures. *International Journal of Entrepreneurial Behaviour & Research*, 23(1), 136–158.

### The present study

Studies that have used the IBI have relied solely on classical higher-order confirmatory factor analysis (CFA). Bifactor modelling offers a plausible alternative to appreciate the dimensionality of the scale. The study used bifactor modelling to provide additional insights into the dimensionality of the IBI. The results inform researchers on whether the IBI scores should be interpreted as one summative score or as sub-scale average scores.

The study also examined the measurement invariance (also known as measurement equivalence) of the IBI between gender groups. Measurement invariance evaluates whether the innovative work behaviour construct, measured using the IBI, is assessed the same way between gender groups (Sanhokwe & Takawira, 2022). Gender differences have been reported or critiqued in extant literature (see De Bruin & Steyn, 2020). Hence, the need to assess the equivalence of the measure to facilitate valid interpretations. Informed by the results of the invariance test, the study assessed whether there was a significant difference in employee innovative behaviour by gender.

## Methods

### Study participants

A probability sample of 102 employees was drawn from a large manufacturing firm in Zimbabwe. The firm has about 800 employees across all levels of work. Female employees constituted 35% of the sample. By age, four in five employees were below the age of 40 years. By level of work, 18% were junior employees; the remainder were senior employees.

### Procedure

Data were collected online using the KoBo Toolbox survey platform (https://www.kobotoolbox.org/). Internal channels were used to broadcast the survey link to all employees. Online data collection has become a vital, plausible alternative for interacting with respondents since the onset of the coronavirus disease 2019 (COVID-19) pandemic in 2019 (Biemer et al., 2021).

### Data analysis

The IBM^®^ SPSS^®^ version 26 software generated the factor loadings as well as the construct reliability and validity-related outputs. The study employed the bifactor indices calculator, accessible at http://sites.education.uky.edu/apslab/resources/ to compute the relevant estimates and indices for ascertaining the factor structure and dimensionality of the IBI (Dueber, 2017). The outputs were interpreted based on thresholds established in the literature (Dueber, 2017). Multi-group CFA was used to test the invariance of the IBI across gender groups. The study used the chi-square test to assess for differences in employee innovative behaviour by gender.

### Ethical considerations

Voluntary participation in the study was satisfied at the organisation and individual employee levels. Employee participation was strictly confidential, safe and voluntary. Although the survey link was shared with all employees, employees could opt out easily by simply not clicking the survey link, thus not investing in the survey. The online survey landing platform provided information about the investigation, including the time to complete the survey. Data collection did not include any personally identifiable information. The employees were free to complete the survey at a convenient time. Contact details were provided to facilitate any follow-ups as needed.

## Results

### Construct reliability and validity

The full scale exhibited internal consistency reliability, that is, Cronbach alpha, *α* = 0.92; see Table 2. The composite reliability (CR) of 0.93 further affirmed the internal consistency of the measure (Reise et al., 2013). The six sub-scales were also internally consistent (see Table 2).

**TABLE 2:** Construct reliability and validity.

Latent variable	Mean	Cronbach’s alpha	Average variance extracted
IBI	5.324	0.916	-
**Sub-scales**	-	-	0.722
Idea generation	5.963	0.841	-
Idea search	4.487	0.929	-
Idea communication	5.573	0.909	-
Implementing starting activities	4.487	0.923	-
Involving others	5.680	0.860	-
Overcoming obstacles	5.755	0.877	-

IBI, Innovative Behaviour Inventory.

The average variance extracted (AVE) estimates the amount of variation that the innovative work behaviour construct explains in the observed 20 variables or items to which it is theoretically related (Farrell, 2010). The AVE for the full scale was greater than the 0.5 threshold, an indication that the construct explains a significant proportion of the observed variance (Sanhokwe, 2022b). In unison, the α, CR and AVE attest to the robust quality of the measure in the study setting.

### Bifactor confirmatory factor analysis

#### Factor-level outputs

The explained common variance (ECV) estimates the amount of variance in the IBI attributed to the general factor. The general factor accounted for 64% of all common variance (see Table 3). The ECV is substantial and indicates that the observed items represent a facet of the same general construct, that is, innovative work behaviour.

**TABLE 3:** Bifactor model level outputs for the IBI scale.

Statistical measure	Value
Percent of uncontaminated correlations (PUC)	0.685
Explained common variance (of general factor)	0.639
Average relative parameter bias (ARPB)	0.123

IBI, Innovative Behaviour Inventory.

#### Model level outputs

Table 3 contains information on three model-level outputs. The percentage of uncontaminated correlations (PUC) is a statistical measure depicting the number of unique correlations among all items of a scale explained only by the general factor (Rodriguez et al., 2016). The average relative parameter bias (ARPB), on the other hand, depicts the distortion that occurs when the items of a measure are forcibly fitted into a unidimensional form (Rogers et al., 2020). According to Reise et al. (2013), when the values of the uncontaminated correlations percentage are lower than 0.80, the general ECV values exceed 0.60, and OmegaH values exceed 0.70; this suggests that the instrument is primarily unidimensional. The results in Table 1 (PUC = 0.685; general ECV = 0.639) suggest that the IBI could be considered unidimensional.

The ARPB (0.123) suggests that forcing all extracted items of the IBI into a unidimensional form poses no significant concern, thus further suggesting the unidimensionality of the measure (Rodriguez et al., 2016).

### Measurement invariance

Three phases of measurement invariance testing were followed, that is, configural, metric and scalar. Invariance tests are hierarchical, that is, stringent constraints are placed, in increasing fashion, on the variable parameters to ascertain if, indeed, there is measurement equivalence (Nouwen et al., 2021). The configural invariance test examined whether the bifactor structure fitted well across gender groups. The bifactor model was fit for the two gender groups, leaving all item intercepts and factor loadings free to vary for each of the two groups.

Metric invariance determined whether all the extracted items of the IBI loaded onto the specified latent factor with similar magnitude between the two groups. All factor loadings were restricted to be equivalent between the gender groups. Only the item intercepts were allowed to vary freely. For scalar invariance, the item intercepts had to be equal across the gender groups.

The literature references several model fit indices for assessing the adequacy of the three models. This study focused on the comparative fit index (CFI), the standardised root mean square residual (SRMR), and the root mean square error of approximation (RMSEA) to minimise the type I and type II errors (Keum et al., 2018). According to Chen (2007), for CFI, values > 0.90 are deemed desirable for a good fit of the model with the study data. The threshold of the SRMR is estimated to be close to 0.08 (Chen, 2007). For the RMSEA, values close to 0.06 are desirable (Chen, 2007).

Table 4 shows the model fit indices for various invariance tests, that is, the metric, scalar and configural. As shown in Table 4, the configural model showed a good fit (CFI values > 0.90; SRMR and RMSEA < 0.08).

**TABLE 4:** Invariance testing.

Variable	CFI	SRMR	RMSEA	90% CI	ΔCFI	ΔSRMR	ΔRMSEA
**Model**							
Configural	0.967	0.071	0.071	0.061 – 0.081	-	-	-
Metric	0.965	0.070	0.063	0.059 – 0.067	-	-	-
Scalar	0.964	0.073	0.062	0.060 – 0.064	-	-	-
**Model comparison**							
Configural vs. metric	-	-	-	-	−0.002	−0.001	−0.008
Metric vs. scalar	-	-	-	-	−0.001	−0.003	−0.001

CFI, comparative fit index; SRMR, standardised root mean square residual; RMSEA, root mean square error of approximation; CI, confidence interval.

Similarly, the model for metric invariance also exhibited adequate fit. The results in Table 4 show that the change in fit indices between the configural and metric models was marginal. The scalar model also showed a good fit for the data (see Table 4). There were minor differences between the metric and the scalar models (ΔSRMR = −0.003; ΔCFI = −0.001; ΔRMSEA = −0.001). Chen (2007) suggests that Δ changes < 0.03 for SRMR, < 0.01 in the CFI and < 0.015 in the RMSEA denote the equivalence of the measure in use. Overall, the results in Table 4 suggest the measurement invariance of the IBI between the gender groups.

### Levels of employee innovative behaviour

The observed levels of employee innovative behaviour were on the high end of the scale, that is, mean = 5.324, 95% confidence interval (CI): 5.084, 5.564. Items such as ‘When something does not function well at work, I try to find new solutions’ and ‘I try to get new ideas from colleagues or business partners’ scored extremely high. On the low end of the spectrum (but above the neutral score) were items such as ‘I develop suitable plans and schedules for the implementation of new ideas’ and ‘I look for and secure funds needed for the implementation of new ideas’. These aspects – at both ends of the spectrum – have significant practical implications for leadership teams that desire high levels of innovation in the workplace.

### Gender and the observed levels of employee innovative behaviour

The chi-square test assessed whether gender reliably differentiated employees’ innovative behaviour. There were significant differences in employee innovative behaviour by gender, *χ*^2^(2, *N* = 102) = 23.045, *p* = 0.000.

## Discussion

The IBI represents an integrated model of the innovative work behaviour construct. Whereas the original study established a second-order model for the IBI, this study modelled the general factor of the IBI. As alluded to earlier, theoretically, innovative work behaviour is a multi-dimensional construct. However, the presence of a strong underlying general factor suggests that the observable items represent a facet of the same construct. The concept of innovative work behaviour pervades and is significantly greater than its constituent elements individually. In unison, the observed items capture the full breadth of the innovative work behaviour construct (Rogers et al., 2020). To our knowledge, this may be the first study to model the general factor of the IBI. The confirmed unidimensionality of the IBI suggests that academic and corporate researchers should interpret the IBI as a single summative score when facilitating workplace studies and related interventions (Sanhokwe, 2022b).

Instruments should exhibit robust evidence of impartiality and fair measurement to avoid potential misuse in the workplace (De Beer et al., 2022). Ascertaining the measurement invariance of the instrument serves as the basis for comparing the means. Making valid inferences presupposes that the IBI measures innovative behaviour in the same way across sub-groups of interest. A comparison of means would have been invalid if measurement invariance could not be ascertained. By confirming the measurement invariance of the IBI, these findings offer corporate researchers and leadership teams an opportunity to facilitate sub-group analysis (Sanhokwe, 2022b). Sub-group analysis allows corporate researchers and leadership teams to tailor their workplace interventions for specific groups to enhance desired behaviour or workplace outcomes.

Different scholars have challenged findings that associate gender with innovative work behaviour (De Bruin & Steyn, 2020). Extant literature suggests that resources and contexts mediate the gender–innovation relationship (De Bruin & Steyn, 2020; also see Fatemi et al., 2021). It is fair to acknowledge that results from extant research on the gender-innovative behaviour nexus have been mixed, calling into question the essence of using the men–women dichotomy. It may be an opportune time to explore other gender-neutral approaches that carry the discipline forward.

This may also be the first study to validate, in a sub-Saharan context, the psychometric properties of the IBI. Effectively assessing the innovation capabilities of employees, on an ongoing basis, across all levels, provides organisations with timely information on the quality of this dynamic capability. When done in an ethically correct manner, using standard measures, these assessments assist organisations in developing systematic, sustainable and targeted interventions that promote the desired behaviour.

Innovation-driven growth demands that organisations consistently assess and develop environments or conditions that support the innovative behaviour of employees. Given its robust qualities, the IBI adds to the suite of available tools for assessing innovative work behaviour and facilitating appropriate interventions. Organisations should invest in appreciating the personal and organisational factors that promote and sustain the behaviour within their specific settings.

### Limitations and areas for future research

Although focusing on a single firm in one industry allowed the context and the situation embedded therein to be interrogated with greater detail while minimising potential external influences on the phenomenon of interest, it also places constraints on external validity. While the sample size was powered enough, it still limits drawing the whole-of-industry conclusions. Furthermore, the study did not translate the instruments to local languages, expectedly so given that English is a medium of communication in most workplaces in the country. However, comprehension differs across levels of work and individuals. This could have constrained the uptake of the survey. Future studies should examine the criterion-related validity of the scale and assess validity of the scale using multi-sector samples. The future demands an interrogation of how innovative work behaviour accumulates at the team and organisational levels. Cross-level, integrative research (using dyads) may satisfy this need.

## Conclusion

The results depict the IBI as a valid and reliable measure of innovative work behaviour. The study modelled the general factor of the IBI. The IBI was invariant between gender groups. There are several commonly referenced measures of innovative work behaviour. The IBI offers practical advantages because of its integrated nature, diagnostic utility and robust psychometric properties. Organisations in the study setting and similar contexts can utilise the IBI to facilitate periodic innovation behaviour-oriented assessments and monitor the execution of dedicated, pro-innovation-driven growth-oriented interventions.
